# Network pharmacology and molecular docking analyses of the Wuwei Mingmu formula indicate that IL-10 and IL-6 are critical targets against experimental autoimmune uveitis in rats

**DOI:** 10.3389/fphar.2025.1614561

**Published:** 2025-10-14

**Authors:** Xing Liang, Junkun Zhang, Fang Yuan, Li Liang, Jie Li, Yajian Duan

**Affiliations:** ^1^ Department of Ophthalmology, Shanxi Bethune Hospital, Shanxi Academy of Medical Sciences, Tongji Shanxi Hospital, Third Hospital of Shanxi Medical University, Taiyuan, China; ^2^ Third Hospital of Shanxi Medical University, Shanxi Bethune Hospital, Shanxi Academy of Medical Sciences, Tongji Shanxi Hospital, Taiyuan, China; ^3^ Department of finance, Shanxi Provincial Cancer Hospital, Taiyuan, Shanxi, China; ^4^ Department of Cardiology, Shanxi Traditional Chinese Medicine Hospital, Shanxi Province Academy of Traditional Chinese Medicine, Taiyuan, China

**Keywords:** network pharmacology, molecular docking, Wuwei Mingmu formula, experimental autoimmune uveitis, non-infectious uveitis, IL-6, IL-10

## Abstract

**Objectives:**

In this study, we analyzed the use of the *Wuwei Mingmu formula* (WMF) for treating experimental autoimmune uveitis (EAU) based on a network pharmacology approach.

**Methods:**

We obtained an integrated gene set between WMF and EAU using the TCMSP database and GeneCards database. Gene Ontology (GO), Kyoto Encyclopedia of Genes and Genomes (KEGG) enrichment analysis, and protein-protein interaction (PPI) network analyses were used to elucidate possible therapeutic mechanisms. Moreover, the relationships among the herbal composition, active ingredients, therapeutic targets, and critical cell signaling pathways used to treat EAU were analyzed. Molecular docking was performed to elucidate the patterns of interactions between the active compounds and the targeted proteins. EAU rat models were constructed to examine the therapeutic efficacy of WMF *in vivo*.

**Results:**

An integrated gene set of 30 genes was acquired. The results of the GO and KEGG analyses indicated that WMF could regulate immune responses and vascular functions during EAU treatment. The PPI network and subnetworks confirmed the presence of 15 hub genes. A network pharmacology map was drawn to understand the complex relationships among herbs, active compounds, targets, and signaling pathways. Additionally, molecular docking was performed on genes with the highest significance (IL-10 and IL-6), representing T-helper activation in immune-mediated uveitis. The active compounds of WMF rapidly docked with IL-10 and IL-6 in the grid box. The results of the *in vivo* assays revealed that WMF treatment significantly attenuated the pathological changes in EAU rat eyes and alleviated the inflammatory response. Moreover, the increase in the level of IL-6 in EAU rats decreased after WMT stimulation, whereas the IL-10 levels increased in EAU rats after WMF treatment.

**Conclusion:**

WMF can be used to treat EAU as it can modulate immune responses and vascular functions. IL-10 and IL-6 are the main therapeutic targets of WMF, and the herbal composition of WMF can be further optimized to increase its therapeutic efficacy.

## 1 Introduction

Non-infectious uveitis (NIU) is one of the leading causes of vision loss and blindness. Researchers often use well-established experimental autoimmune uveitis (EAU) animal models. These models help explore the underlying mechanisms and potential therapeutic interventions for this complex human disease. The immune response in EAU is primarily mediated by T cells and leads to overactivation of cytokines such as interleukins, interferons, and vascular endothelial growth factor (VEGF) in ocular tissues and peripheral blood (Caspi, 2003; Sun et al., 2018; Shome et al., 2023; Goldhardt and Rosen, 2016). Therefore, EAU treatment focuses on rapidly and effectively controlling inflammation while suppressing excessive autoimmune reactions. The primary objectives in EAU treatment are to control inflammation, inhibit excessive autoimmune responses, reduce drug side effects, prevent further damage to eye tissues, and protect visual function.

The 5 main classes of drugs used to treat EAU include corticosteroids, T-cell immunosuppressants, antimetabolites, alkylating agents, and biologics (Knickelbein et al., 2017). These drugs can be administered locally to ocular tissues or systemically, and treatment may follow a step therapy approach (Foster et al., 2016). Although the efficacy for treating this disease has significantly improved over time, adverse effects such as hyperglycemia, systemic hypertension, a decrease in bone mineral density (BMD), depression, and weight gain are often reported (Carnahan and Goldstein, 2000). Therefore, the treatment regimen needs to be optimized.

Traditional Chinese medicine (TCM) is recommended for treating Non-infectious uveitis in China due to its holistic approach to treating diseases with complex pathological mechanisms, including EAU. However, the components of TCM are highly complex, and their pharmacological mechanisms are challenging to elucidate. Additionally, clinical efficacy evidence remains limited. Recently, with the development of network pharmacology, systems biology methods have been applied to analyze the molecular mechanisms underlying the pharmacological effects of herbal medicines. These data-driven approaches help answer unresolved questions and reveal new research opportunities (Hopkins, 2007; Hopkins, 2008; Lin et al., 2021; Xia et al., 2020; Ru et al., 2014).

In this study, we applied a network pharmacology approach to analyze a TCM formula, known as WMF, which is used clinically at Bethune Hospital of Shanxi Province, China, to treat non-infectious uveitis. The composition of the WMFincludes *Cassia obtusifolia* L. *(Juemingzi*, JMZ*), Paeonia lactiflora Pall. var. Trichocarpa (Chishao*, CS*), Atractylodes lancea (Thunb.) Dc. (Cangzhu,* CZ*), Lycium barbarum* L. *(Gouqizi*, GQZ*)and Astragalus complanatus* R. Br. *(formerly referred to as Astragali Complanati Semen, Shayuanzi*, SYZ). Based on the findings of this study, we can identify the effective targets of TCM components for treating EAU, analyze the mechanisms underlying their pharmacological effects at the molecular level, and validate the binding interactions between selected targets and active components using molecular docking technology simulations.

## 2 Materials and methods

### 2.1 Identification of active compounds and target genes of WMF

We acquired the active compounds and target genes of WMF through the Traditional Chinese Medicine Systems Pharmacology (TCMSP) database (https://www.tcmsp-e.com/). The database comprises 499 types of Chinese herbs registered in the Chinese pharmacopeia and provides essential *in silico* absorption, distribution, metabolism, and excretion (ADME)-related properties, as well as the drug targets and associated diseases of each active compound. We investigated the chemical compounds by retrieving the name of each herb in WMF, including *Juemingzi, Chishao, Cangzhu, Gouqizi,* and *Shayuanzi.* Next, we filtered the pharmacokinetic indices for active compounds whose oral bioavailability was greater than 30% and whose drug-likeness was greater than 0.18. The corresponding target proteins of the active compounds were screened using the TCMSP database. Subsequently, the target proteins were converted into standard gene symbols using UniProt (http://UniProt.org).

### 2.2 Therapeutic genes for EAU

To identify therapeutic genes relevant to non-infectious uveitis, we utilized the GeneCards database a comprehensive, searchable, and integrated resource consolidating gene-centric information from over 150 sources (https://www.genecards.org/). Since our TCM formula is specifically designed to treat this condition, we used “non-infectious uveitis” as the search keyword. This choice reflects the experimental context, as EAU models are widely employed in animal studies of the disease. By focusing on the human disease terminology, we maintain consistency with clinical relevance and align with the majority of publications in this field.

### 2.3 Integrated gene set between WMF and EAU

The overlapping gene set between WMF target genes and EAU therapeutic genes was obtained by intersecting these 2 gene lists. This process was performed using the online tool Venny 2.1 (http://bioinfogp.cnb.csic.es/tools/venny/).

### 2.4 Enrichment analysis

The DAVID database version 6.8 (https://david.ncifcrf.gov/) and the KEGG database (Kyoto Encyclopedia of Genes and Genomes, http://www.kegg.jp/) were used to perform enrichment analysis, including Gene Ontology (GO) functional analysis—Biological Process (BP), Cellular Component (CC), and Molecular Function (MF) —and KEGG pathway analysis. The species was limited to *Homo sapiens*. The threshold value was set as *p* < 0.05. This process was implemented by applying the Org. Hs.eg.db (Version 3.8.2) and ClusterProfiler (Version 3.9) packages in R software version 4.0.3.

### 2.5 Pharmacological network of active compound, target genes, and keysignaling pathways

Based on the active compounds of WMF, the overlapping gene set, and KEGG pathway analysis, we constructed a network integrating active compounds, their target genes, and signaling pathways using Cytoscape version 3.6.1.

### 2.6 Analysis of the PPI network

The STRING database was used to construct the PPI network. The integrated gene set between WMF and EAU was submitted to the STRING database online. We selected *H. sapiens* as the organism. The minimum required interaction score was set to moderate confidence (0.400). The PPI network image was downloaded in PNG format from the STRING database for visualization. The network data were then imported into Cytoscape version 3.6.1. Following the MCC method, the core subnetworks were identified using the MCC method within theCytoHubba plugin.

### 2.7 Molecular docking

Computational analysis predicted that Quercetin (3,3′,4′,5,7-pentahydroxyflavone; TCMSP ID: MOL000098), wogonin (5,7-dihydroxy-8-methoxyflavone; TCMSP ID: MOL00173), and paeoniflorin (TCMSP ID: MOL001924). directly interact with IL-6 or IL-10. Three-dimensional crystal structures of human IL-6 (PDB ID: 1ALU) and IL-10 (PDB ID: 2ILK) were obtained from the RCSB Protein Data Bank (https://www.rcsb.org/). Subsequently, three-dimensional ligand structures were obtained from the TCMSP database (https://www.tcmsp-e.com/).

All docking calculations were performed with AutoDock 4.2. Briefly, water molecules and heteroatoms were removed from the protein structures. Polar hydrogens and partial Gasteiger charges were added, and non-polar hydrogens were merged using AutoDockTools (ADT, version 1.5.6). Rotatable bonds of the ligands were defined with AutoDockTools (ADT). A grid box (60 × 60 × 60 Å with 0.375 Å spacing) was centered on the active site of each protein, which was determined based on known binding site residues. The Lamarckian genetic algorithm (GA) was employed with 10 independent runs for each complex. The lowest-energy conformation for each ligand-protein complex was selected as the final docking pose. Binding affinities were expressed as binding energies in kcal/mol.

### 2.8 Preparation and administration of WMF

The crude herbal materials of Juemingzi, Chishao, Cangzhu, Gouqizi, and Shayuanzi were purchased from the Pharmacy of Shanxi Traditional Chinese Medicine Hospital, with all herbs authenticated by the pharmacy. The herbs were combined at a fixed mass ratio of 3:3:2:3:3 and subjected to aqueous decoction. The resulting decoction was filtered, concentrated under reduced pressure, and lyophilized to obtain a dry powder. To ensure experimental consistency throughout the study, a single, large batch of the freeze-dried powder was prepared at the outset. This entire batch was then portioned into single-use aliquots for the entire duration of the animal experiments. This strategy effectively eliminates inter-batch variability and guarantees consistent chemical exposure for all subjects. The aliquots were stored in light-resistant glass vials at −80 °C until use. For administration, the powder was reconstituted in distilled water to form a suspension at a concentration equivalent to 0.315 g of crude herb per milliliter. The total daily dose was administered to rats via oral gavage in two divided doses.

### 2.9 Animal experiments

This study protocol was approved by the Ethics Committee of Shanxi Bethune Hospital. Healthy Lewis rats (*Rattus norvegicus*) (6–8 weeks old, 160–180 g), with equal numbers of males and females, were obtained from Beijing Vital River Laboratory Animal Technology Co., Ltd. [license No.: SCXK (Beijing) 2021-0006]. Rats were randomly assigned to three groups (n = 8 per group): the Sham group, the EAU group, and the EAU + WMF group. To establish the EAU model, an emulsion was prepared by mixing interphotoreceptor retinoid-binding protein (IRBP) peptide and complete Freund’s adjuvant (CFA; Sigma-Aldrich) at a 1:1 (v/v) ratio. The mixture was supplemented with 5 mg/mL heat-inactivated *Mycobacterium tuberculosis* H37Ra (ATCC, United States) and then emulsified in PBS to a final volume of 300 μL. All anesthetized via intraperitoneal injection of sodium pentobarbital (80 mg/kg). Subsequently, for immunization, rats in the EAU and EAU + WMF groups received approximately 100 μL of the IRBP-containing emulsion, equally divided among 5 subcutaneous injection sites: both hind footpads (10 μL per site), both flanks (20 μL per site), and the dorsal midline (40 μL). Rats in the Sham group received injections of an identical emulsion without IRBP at the same sites and volumes.

To evaluate the effects of WMF on experimental autoimmune uveitis (EAU), rats in the EAU + WMF group received a daily oral gavage of WMF suspension at a dose of 630 mg/kg. This dose was derived from the human clinical dose of 70 g per day (for a 60 kg adult) converted according to body surface area using a standard conversion factor of 0.018, equivalent to 1.26 g per day for a 200 g rat (630 mg/kg). Rats in the EAU control group were administered an equal volume of normal saline daily via oral gavage.

Following immunization, clinical signs of anterior segment inflammation in rats were monitored using a Genesis-D camera. The clinical manifestations were then scored on a scale from 0 (normal) to 4 (severe inflammation), according to the grading criteria established by Caspi et al. (which comprises a total of 6 distinct grades), as detailed in Table 1. On day 18 post-immunization, pupils were dilated with 0.5% tropicamide eye drops, photographed, and then the rats were euthanized.

**TABLE 1:** Inflammatory grading criteria for rat EAU.

Score	Inflammatory manifestations
0	No inflammation; normal retinal red reflex
0.5	Mild dilation and congestion of iris vessels
1	Moderate dilation and congestion of iris vessels, with miosis
2	Slight anterior chamber turbidity, with diminished retinal red reflex
3	Severe congestion of iris, moderate anterior chamber turbidity, still visible pupil, and dim retinal red reflex
4	Severe congestion of iris, severe anterior chamber turbidity or hypopyon, closed pupil, absence of retinal red reflex, and proptosis

To balance tissue requirements with statistical power, tissue samples from eight animals within each experimental group were pooled in equal amounts. This homogenized pool was subsequently divided equally into three aliquots (hereafter termed composite samples), thereby generating three technical replicates representing the pooled sample of the experimental group.

Each of the three composite samples generated per experimental group underwent quantitative reverse transcription-polymerase chain reaction (qRT-PCR), enzyme-linked immunosorbent assay (ELISA), and Western blot analysis.

To minimize technical variation and enhance measurement precision, each analytical assay (qRT-PCR, ELISA, Western blot) was performed in triplicate on each composite sample. The average of the three technical replicates for each composite sample was calculated, representing the final quantitative value for that biological replicate.

### 2.10 Histopathological analysis

The rats were anesthetized with pentobarbital (50 mg/kg) and sacrificed 18 days after immunization. Their eyes were excised and fixed in 4% paraformaldehyde solution for 24 h in a 12-well plate. After fixation, the eyes were dehydrated, embedded in paraffin, and sectioned at 4 μm thickness. Subsequently, these sections were stained use hematoxylin and eosin (Beyotime, Shanghai, China). Finally, the images were obtained under a light microscope at 200 × magnification. Histopathological changes in the retina were scored on a scale of 0–4 according to the established grading system for EAU (Caspi, 2003), which comprises a total of 6 distinct grades, where 0 represents no inflammation and 4 represents full-thickness retinal destruction (Table 2). Histopathological evaluation was independently performed by two certified pathologists under blinded conditions. The images were scored separately by both evaluators, with any discrepancies resolved through consultation to reach a consensus.

**TABLE 2:** Scoring EAU histopathologically in the rat.

Grade	Area of retinal section affected	Criteria
0	None	No disease, normal retinal architecture
0.5	<1/4	Mild inflamatory cell infiltration of the retina with or without photoreceptor damage
1	≥1/4	Mild inflammation and/or photoreceptor outer segment damage
2	≥1/4	Mild to moderate inflammation and/or lesion extending to the outer nuclear layer
3	≥1/4	Moderate to marked inflammation and/or lesion extending to the inner membrane layer
4	≥1/4	Severe inflammation and/or full-thickness retinal damage

### 2.11 ELISA

The concentrations of inflammatory cytokines (IL-10 and IL-6) in the lymph node, spleen, and eye samples were determined by ELISA. Briefly, the samples were homogenized in RIPA lysis buffer and centrifuged at 2000 *g* for 10 min at 4 °C. The supernatant was collected, and levels of IL-10 and IL-6 were assessed using commercial Rat Interleukin 10 (IL-10) ELISA Kit (F3071-A, FANKEW, Shanghai, China) and Rat Interleukin 6 (IL-6) ELISA Kit (F3066-A, FANKEW, Shanghai, China) in accordance with the manufacturer’s instructions. Absorbance was measured using a DR-200Bs microplate reader (Diatek).

### 2.12 RT-qPCR

TRIpure Total RNA Extraction Reagent (ELK Biotechnology, Wuhan, China) was used for total RNA isolation, and cDNA was subsequently synthesized using EntiLink™ first Strand cDNA Synthesis Super Mix (ELK Biotechnology). PCR analysis was conducted using EnTurbo™ SYBR Green PCR SuperMix (ELK Biotechnology) on a QuantStudio 6 Flex System (Life Technologies). The 2^(-ΔΔCt)^ method was used to quantify relative gene expression, and ACTIN was used as an internal reference. The sequences of primers used in this study are as follows.

IL-10: sense: 5′- ACT​GCT​ATG​TTG​CCT​GCT​CTT​AC-3′,
antisense: 5′-CAG​TAA​GGA​ATC​TGT​CAG​CAG​TAT​G-3’;
IL-6: sense: 5′-TTC​TCT​CCG​CAA​GAG​ACT​TCC-3′,
antisense: 5′-GTG​GGT​GGT​ATC​CTC​TGT​GAA​G-3’;
ACTIN: sense: 5′-CGT​TGA​CAT​CCG​TAA​AGA​CCT​C-3′,
antisense: 5′-TAG​GAG​CCA​GGG​CAG​TAA​TCT-3’.

### 2.13 Western blotting

The concentrations of inflammatory cytokines (IL-10 and IL-6) in the lymph node, spleen, and eye samples were determined by Western blot analysis using specific antibodies from Santa Cruz Biotechnology (IL-6 Antibody, sc-57315; IL-10 Antibody, sc-365858). The spleen, lymph nodes, and eye tissues of the rats preserved at −80 °C were collected, and total protein was extracted from the tissues after treatment with RIPA lysis buffer. The proteins were quantified, separated by electrophoresed, and transferred to a membrane. The membrane was incubated at room temperature with blocking buffer for 3 h, after which it was incubated with antibodies against RORγt (1:1000), Foxp3 (1:1000), RhoA (1:1000), ROCK1 (1:1000), and GAPDH (1:1000) at 4 °C overnight. HRP-conjugated goat anti-rabbit secondary antibodies (1:2000) were added to the membrane, which was incubated at room temperature for 3 h. After the protein bands were developed with an ECL reagent, gray values of the protein bands were analyzed using the ImageJ software.

### 2.14 Statistical analysis

All statistical analyses were conducted using SPSS 25.0. Normality of the distribution was assessed with the Shapiro–Wilk test (appropriate for small samples), and homogeneity of variances was evaluated with Levene’s test. Continuous data were presented as mean ± standard deviation (mean ± SD). Differences among multiple groups were analyzed by one-way analysis of variance (ANOVA). When ANOVA indicated a statistically significant difference, Tukey’s HSD *post hoc* test was applied for pairwise comparisons. The overall comparison among multiple groups was analyzed using the Kruskal–Wallis test, followed by Dunn’s test for multiple comparisons. The significance level was set at α = 0.05. To visualize statistical differences, graphs were generated using GraphPad Prism 10.0. Alternatively, online plotting tools from Xiantao Academic (www.xiantao.love) were also utilized for their accessibility and ease of use. Statistical significance using asterisks: *p < 0.05, **p < 0.01, ***p < 0.001, ****p < 0.0001. The asterisk notation used is defined in the legend of each figure.

## 3 Results

### 3.1 Screening the active compounds and therapeutic targets

**FIGURE 1:**
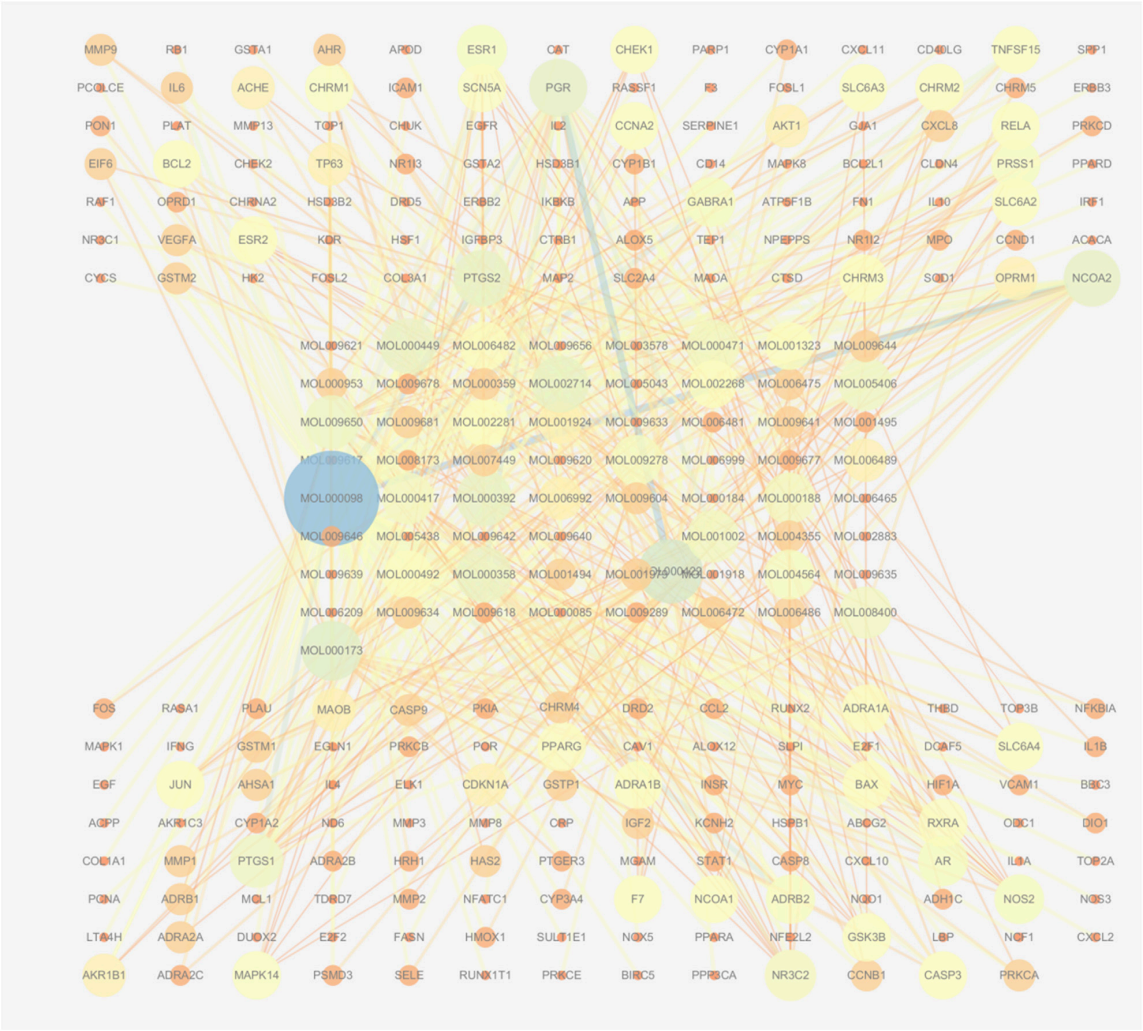
Network of active compounds and their predicted targets in WMF. The central circular nodes represent active compounds. The upper and lower circular nodes represent predicted targets. Edges represent compound-target interactions. Node size is proportional to the degree of connectivity; edge color reflects betweenness centrality.

**FIGURE 2:**
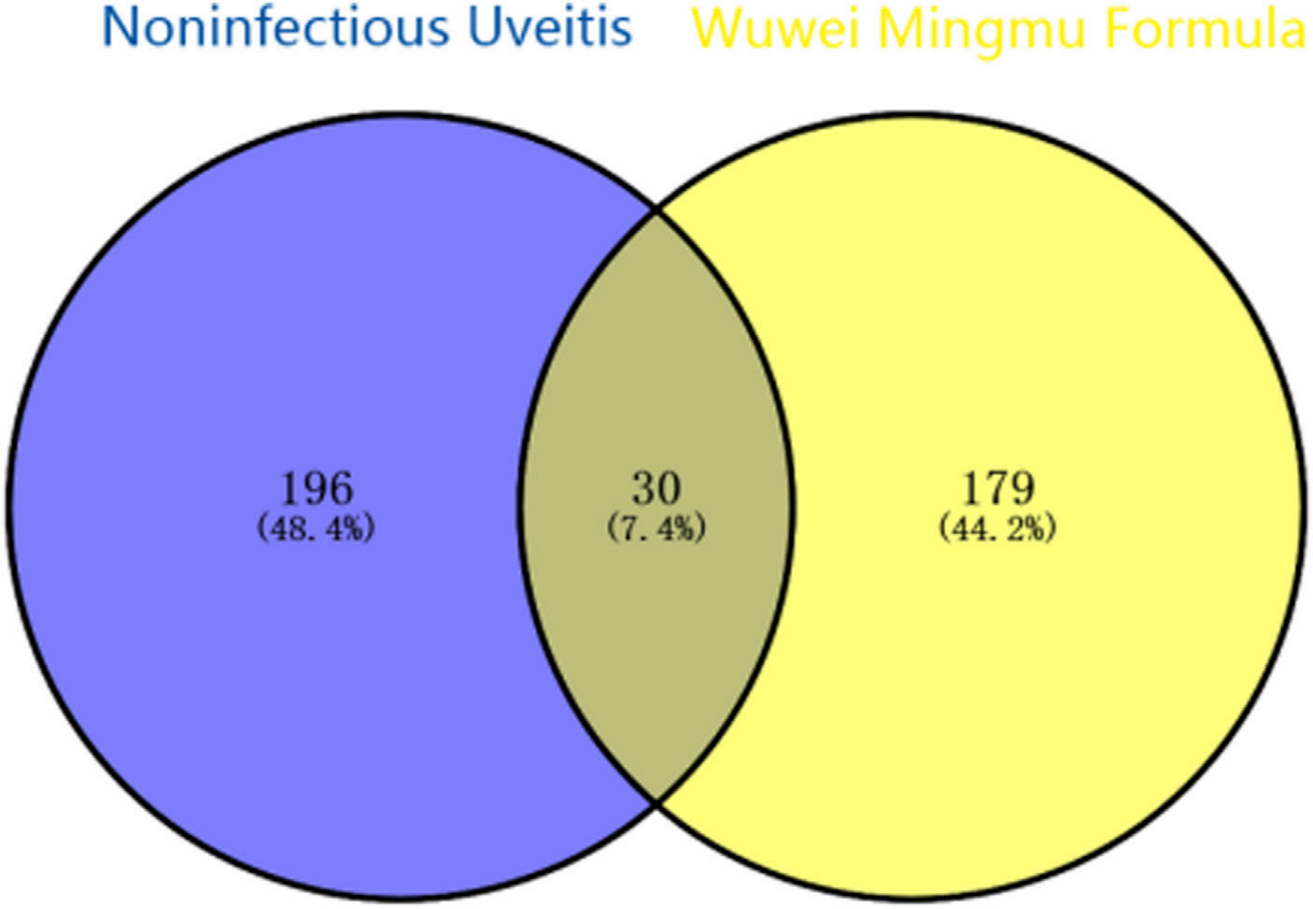
Venn diagram identifying the overlapping targets between WMF and NIU.

**FIGURE 3:**
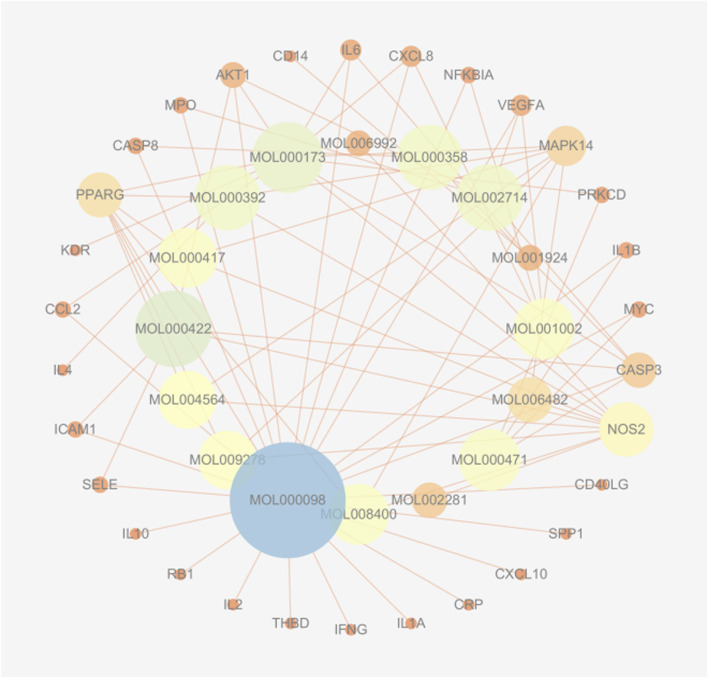
Herb-compound-target network for WMF in EAU treatment. The network exhibits a concentric layout, with inner-ring nodes representing active compounds and outer-ring nodes representing target proteins. Edges connect compounds to their respective targets. The size of a node is proportional to its degree of connections within the network.

Using the TCMSP database, we obtained 65 active compounds after removing duplicates. These compounds were then combined with the search results (Figure 1). We identified 209 related targets associated with these compounds (Figure 1). Additionally, 226 non-infectious uveitis-related genes were obtained from GeneCards database. We determined the integrated gene set representing the intersection of gene targets related to WMF and EAU using Venny 2.1 software. The integrated gene set comprised 30 genes (Figures 2, 3). 16 active compounds were identified as potentially treating EAU (Figure 3).

### 3.2 GO enrichment analysis

**FIGURE 4:**
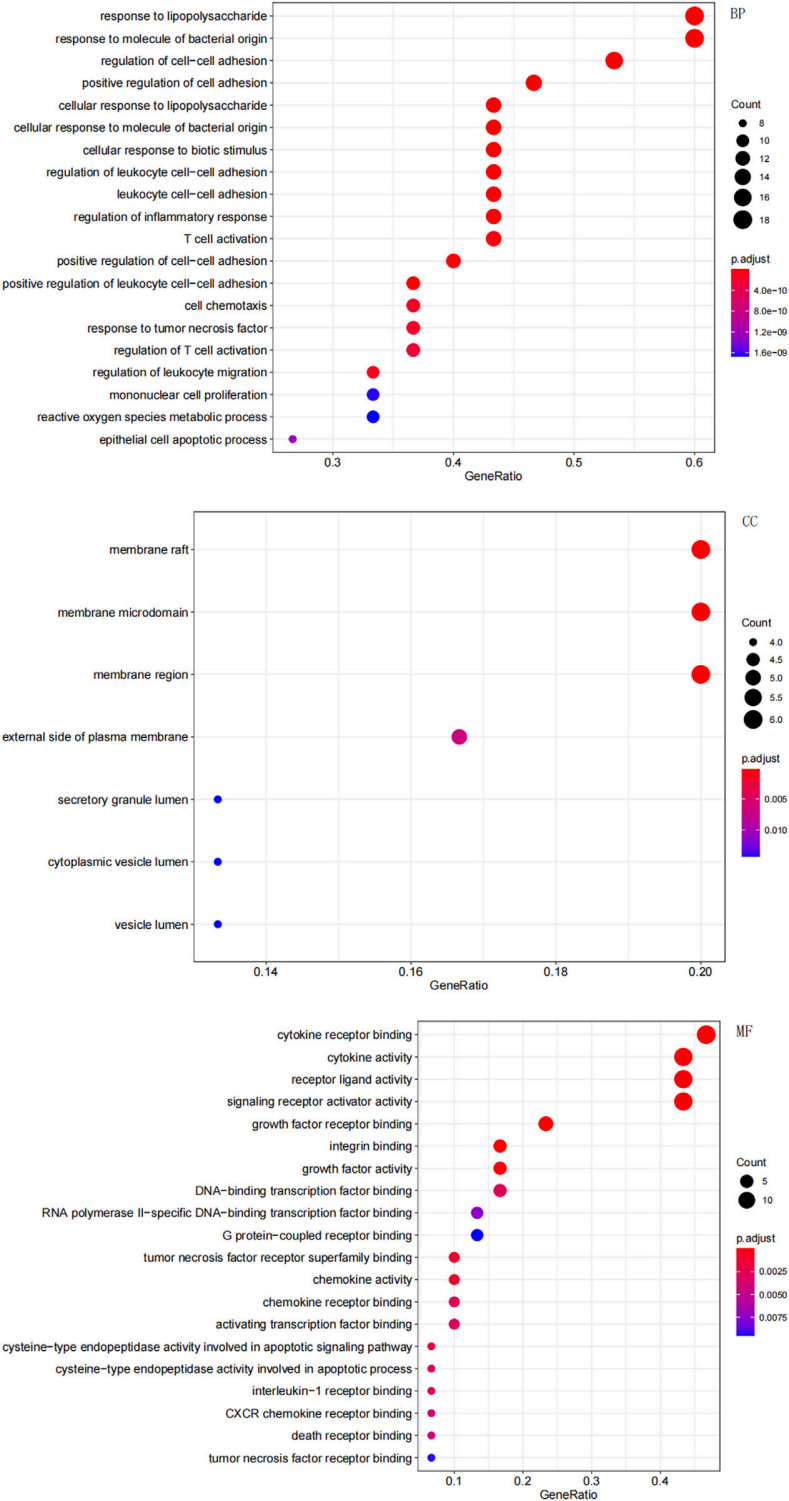
Gene Ontology (GO) enrichment analysis of the overlapping targets. The top significantly enriched terms in biological process (BP), cellular component (CC), and molecular function (MF) are shown. The gene ratio represents the proportion of enriched genes per term. Point size indicates the number of genes enriched in each term.

In total, GO enrichment analysis was performed on the 30 genes. We identified 1,441 significantly enriched GO terms. The top GO terms are shown in Figure 4. The results indicated that the 30 genes were enriched the following categories.

• BP: response to lipopolysaccharide, T-cell activation, and regulation of the inflammatory response;
• CC: membrane rafts, membrane microdomains, and membrane regions;
• MF: cytokine receptor binding and cytokine activity.

### 3.3 KEGG enrichment analysis

**FIGURE 5:**
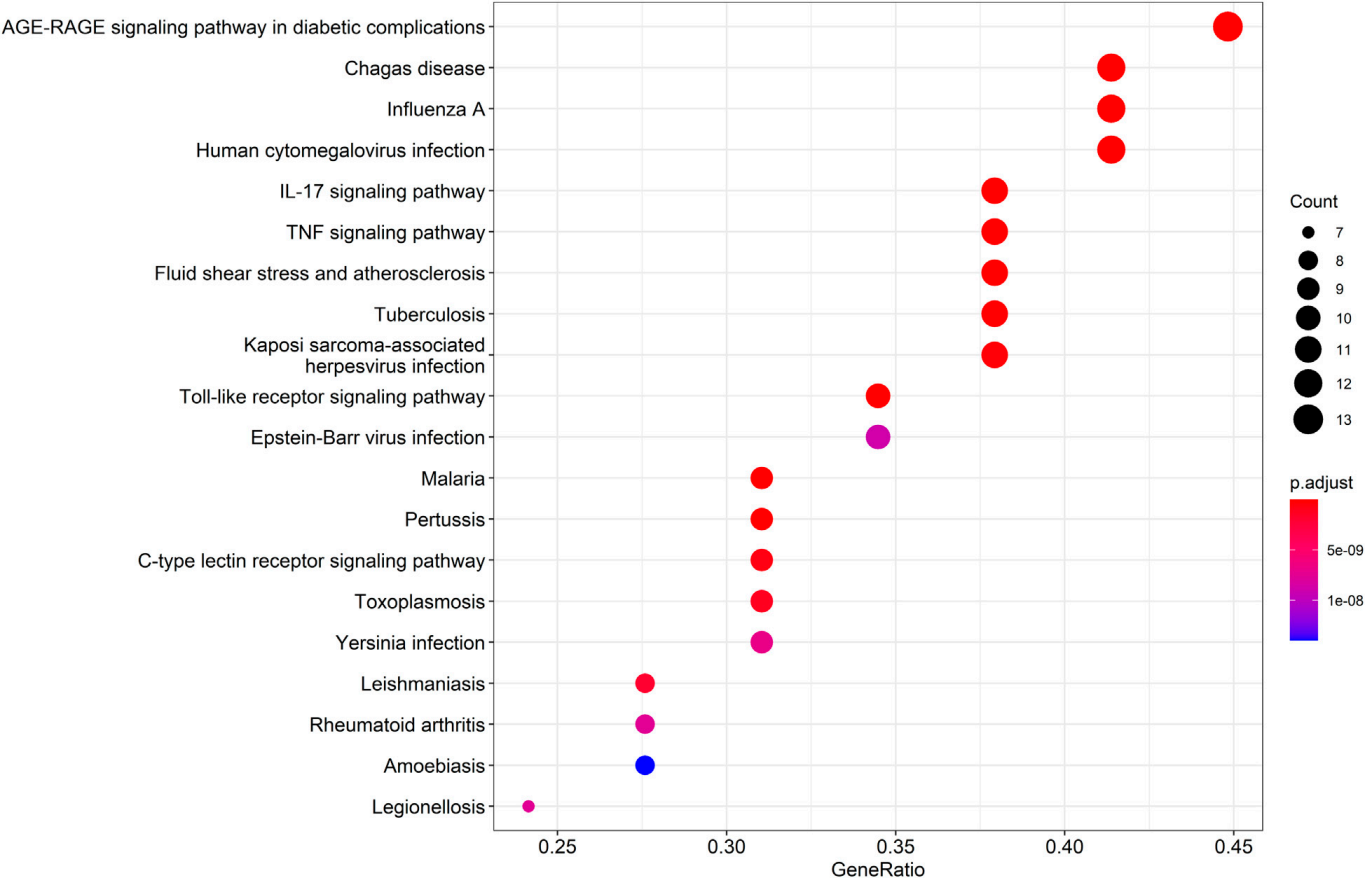
Kyoto Encyclopedia of Genes and Genomes (KEGG) pathway enrichment analysis of the overlapping targets. The top 20 significantly enriched pathways are displayed. The rich factor represents the ratio of enriched genes to the total number of genes in the pathway. Point color and size correspond to the adjusted p-value and the number of enriched genes, respectively.

**FIGURE 6:**
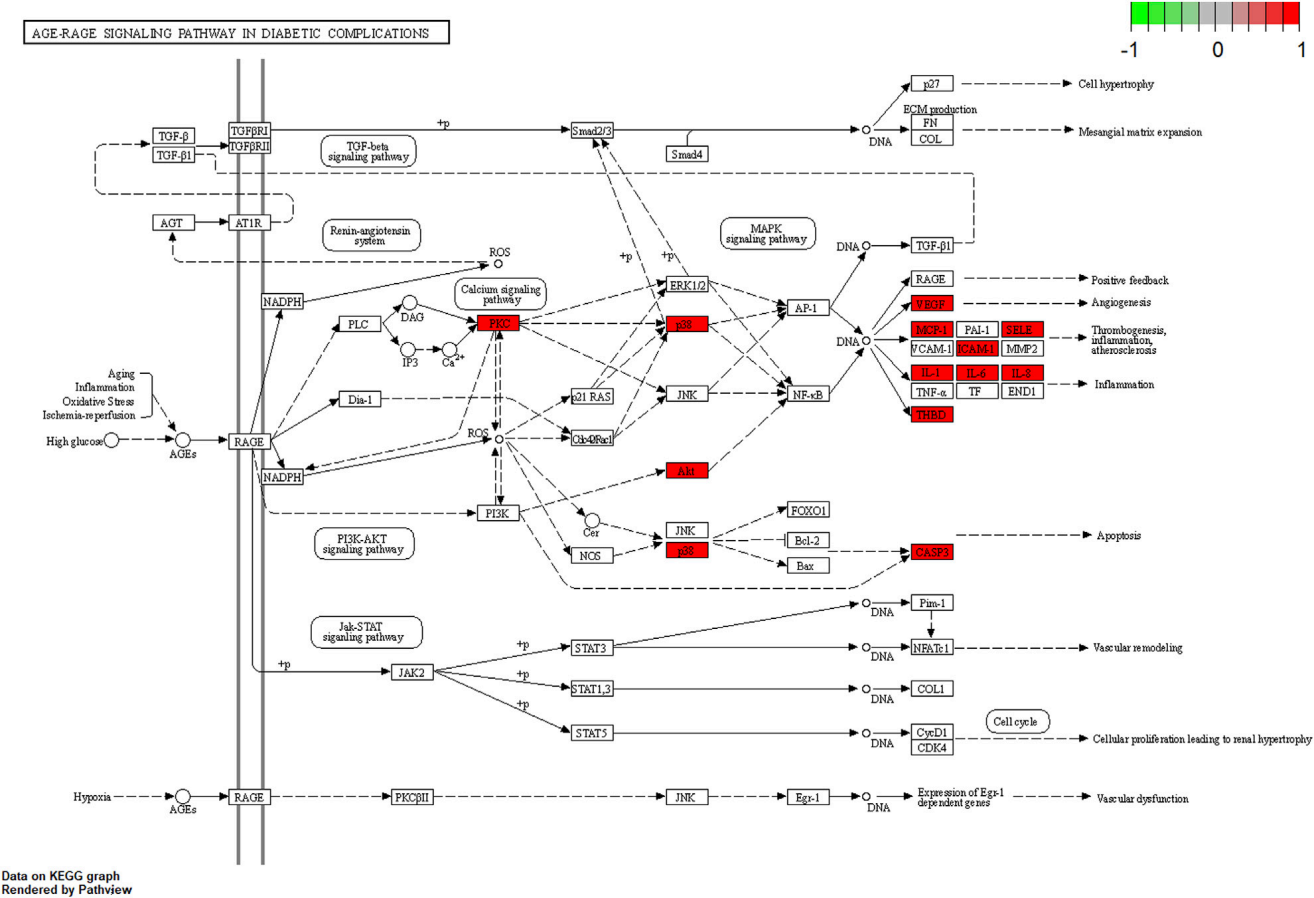
Schematic of the AGE-RAGE signaling pathway in diabetic complications (adapted from KEGG). Genes highlighted in red were among the overlapping targets identified in this study.

KEGG pathway enrichment analysis was employed to clarify the key signaling cascades through which the 30 overlapping targets of WMF mediate therapeutic effects in EAU. These targets were significantly enriched in 124 pathways (*p* < 0.05) (Figure 5) and could be grouped into 2 major functional clusters: (1) immune-inflammatory pathways, exemplified by IL-17, TNF, and Toll-like receptor signaling pathways; and (2) pathways related to vascular dysfunction, highlighted by the involvement of the AGE-RAGE axis (Figure 6). Importantly, IL-6 and IL-10 were common to both clusters and represented 2 pivotal targets whose strong binding affinity to WMF constituents was predicted by molecular docking. Collectively, WMF alleviated EAU by concurrently modulating inflammatory cascades and vascular dysfunction, the two central pathological drivers of the disease. We also provided the IDs and names of crucial signaling pathways in Table 3.

**TABLE 3:** Key signaling pathways enriched by the 30 targets.

ID	Description	GeneRatio	*p*value	*p*.Adjust	*q*value	Count
hsa04933	AGE-RAGE signaling pathway in diabetic complications	13/29	4.08E-18	6.82E-16	2.11E-16	13
hsa05142	Chagas disease	12/29	3.63E-16	3.03E-14	9.36E-15	12
hsa04657	IL-17 signaling pathway	11/29	8.37E-15	4.66E-13	1.44E-13	11
hsa05144	Malaria	9/29	5.64E-14	2.05E-12	6.32E-13	9
hsa04668	TNF signaling pathway	11/29	6.13E-14	2.05E-12	6.32E-13	11
hsa05164	Influenza A	12/29	2.06E-13	5.74E-12	1.77E-12	12
hsa05418	Fluid shear stress and atherosclerosis	11/29	6.90E-13	1.65E-11	5.08E-12	11
hsa04620	Toll-like receptor signaling pathway	10/29	1.30E-12	2.72E-11	8.40E-12	10
hsa05133	Pertussis	9/29	3.03E-12	5.61E-11	1.73E-11	9
hsa05163	Human cytomegalovirus infection	12/29	5.49E-12	9.17E-11	2.83E-11	12
hsa05152	Tuberculosis	11/29	1.20E-11	1.82E-10	5.61E-11	11
hsa05167	Kaposi sarcoma-associated herpesvirus infection	11/29	2.56E-11	3.56E-10	1.10E-10	11
hsa04625	C-type lectin receptor signaling pathway	9/29	5.48E-11	7.04E-10	2.17E-10	9
hsa05145	Toxoplasmosis	9/29	1.08E-10	1.28E-09	3.97E-10	9
hsa05140	Leishmaniasis	8/29	1.70E-10	1.89E-09	5.85E-10	8
hsa05135	*Yersinia* infection	9/29	6.63E-10	6.92E-09	2.14E-09	9
hsa05323	Rheumatoid arthritis	8/29	7.94E-10	7.59E-09	2.34E-09	8
hsa05134	Legionellosis	7/29	8.18E-10	7.59E-09	2.34E-09	7
hsa05169	Epstein-Barr virus infection	10/29	1.00E-09	8.82E-09	2.72E-09	10

### 3.4 The herb–compound–target–critical signaling network

**FIGURE 7:**
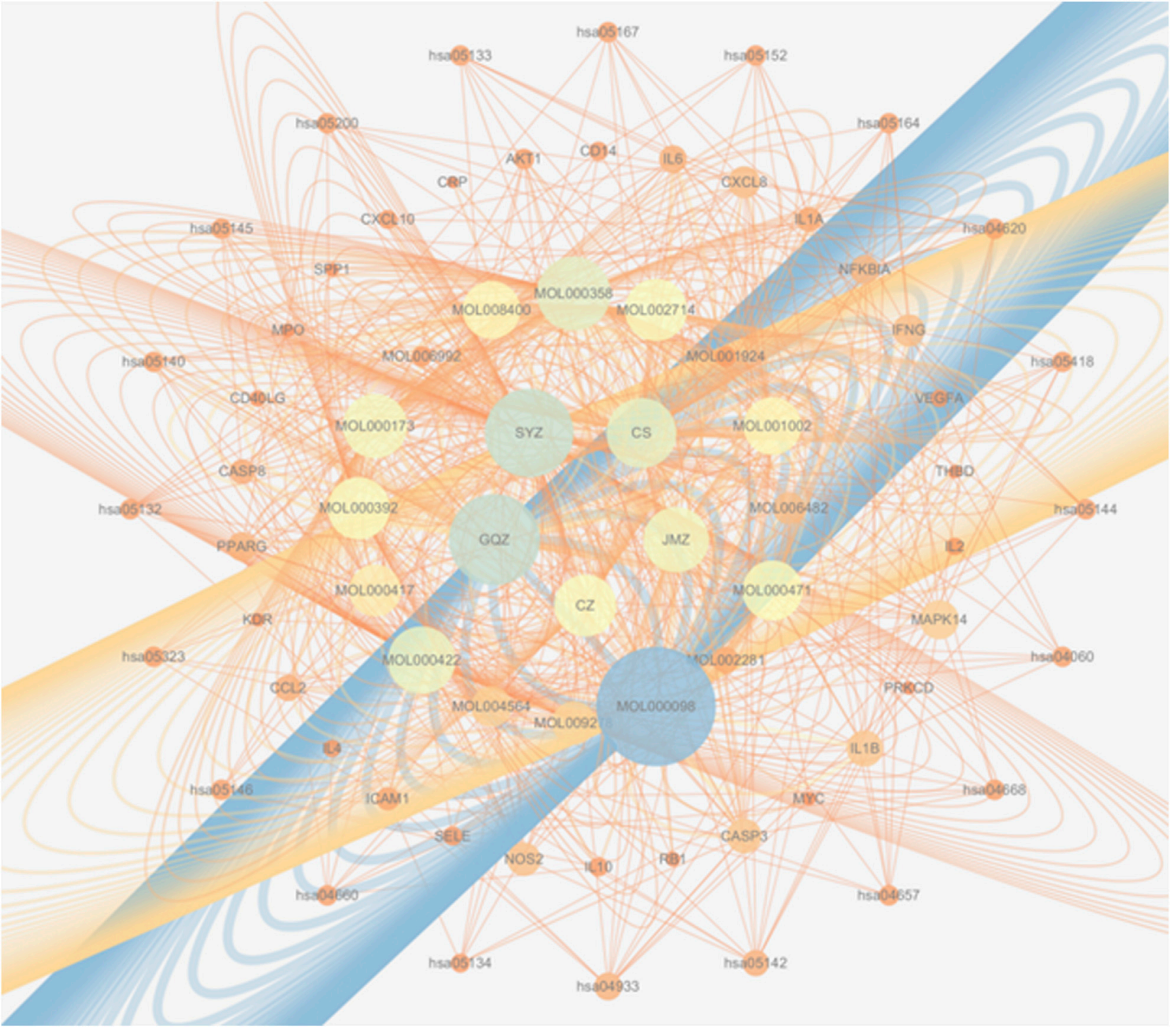
A multi-scale network map of WMF’s action. This integrated network visualizes the therapeutic mechanism across four levels: herbs → compounds → targets → pathways, arranged in concentric circles from the center outward. Node size represents the degree of connectivity within the network.

Figure 7 presents an integrated “herb–active compound–key target–core pathway” network, comprising 5 herbs: *Juemingzi* (JMZ), *Chishao* (CS), *Cangzhu* (CZ), *Gouqizi* (GQZ), and *Shayuanzi* (CZ); 16 active compounds; 30 overlapping targets; and 20 major KEGG pathways. Node size reflects degree, which is defined as the number of connections, while edge color indicates betweenness centrality. Notably.

• SYZ and GQZ display the largest nodes, indicating that their constituents interact with 26–27 targets each and target all 15 hub proteins.
• Central nodes IL-6 and IL-10 are directly linked to multiple inflammatory and vascular pathways, including IL-17, TNF, and AGE-RAGE.

This visualization highlights the multi-component, multi-target, and multi-pathway characteristics of WMF, thereby providing a theoretical basis for subsequent mechanistic validation.

### 3.5 Analysis of the PPI network

**FIGURE 8:**
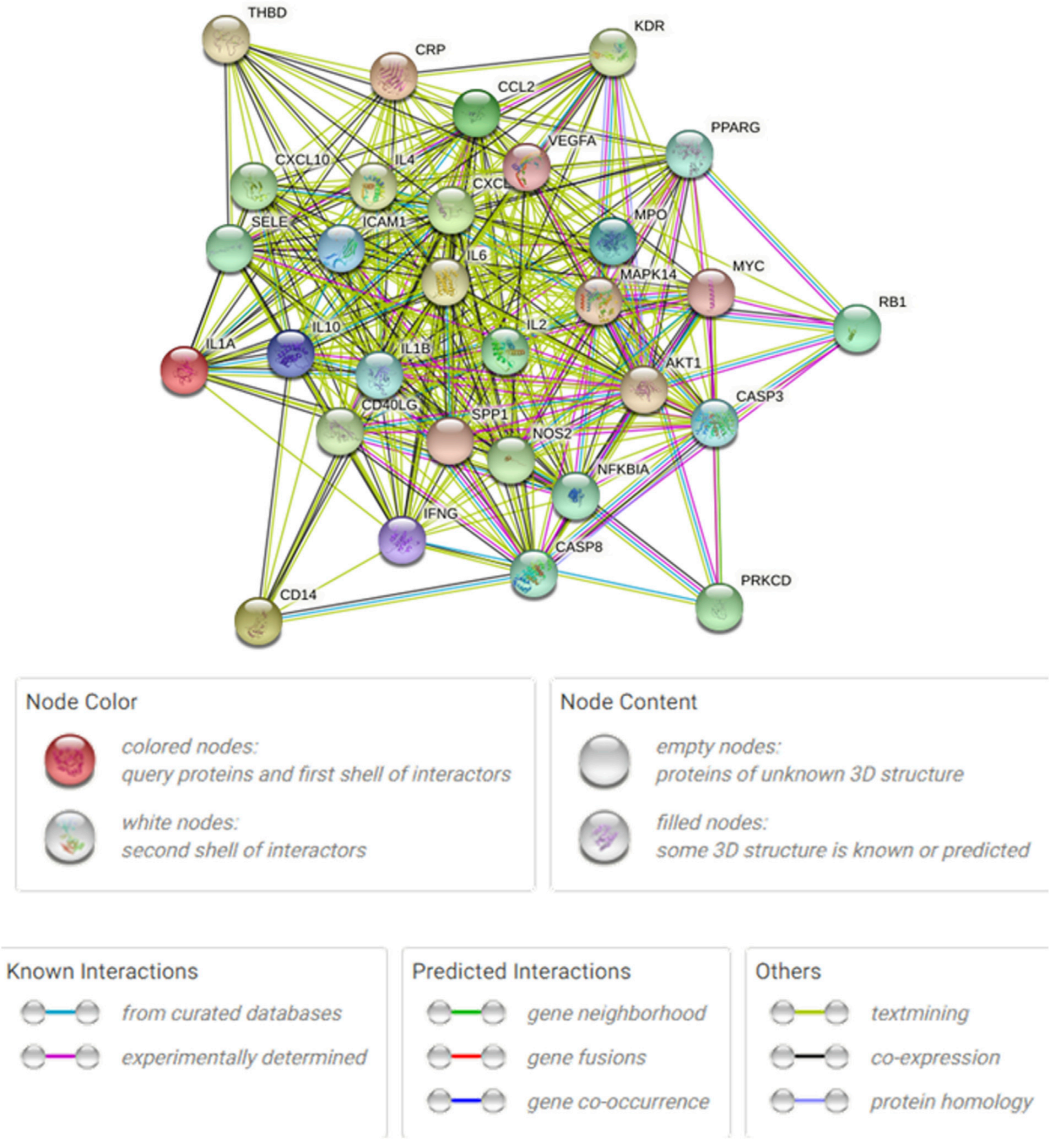
Protein-protein interaction (PPI) network of the 30 overlapping targets. The network was constructed using the STRING database (confidence score ≥0.4). Nodes represent proteins, and edges represent predicted functional associations.

**FIGURE 9:**
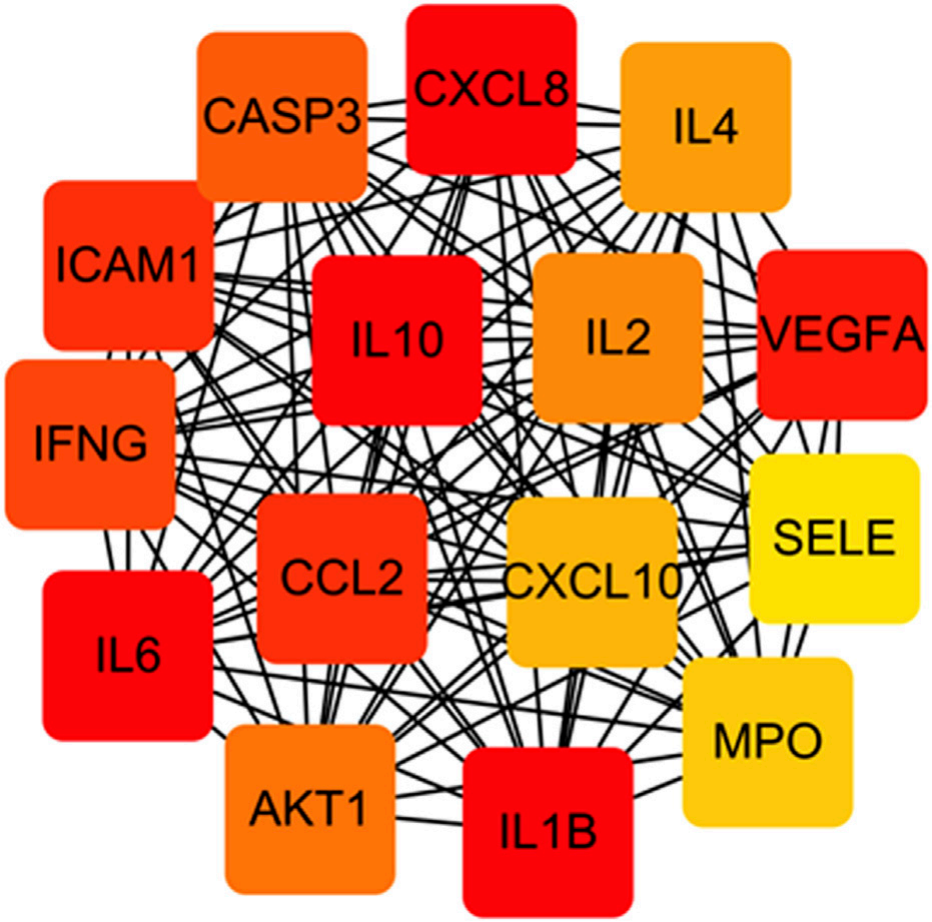
Identification of hub genes from the PPI network. The top 15 hub genes were identified using the CytoHubba plugin with the MCC algorithm. Node color intensity corresponds to the rank order of importance.

Figure 8 shows the overall protein–protein interaction network of the 30 overlapping targets (obtained from STRING with confidence ≥0.4). Figure 9 further extracts the top 15 hub genes using the CytoHubba plug-in, employing the MCC algorithm, and lists their ranks in Table 4. IL-6, IL-10, CXCL8, IL-1β, and other high-degree nodes occupy the central part of the network. This suggests that these hub genes may play pivotal regulatory roles during WMF intervention in EAU.

**TABLE 4:** The rank of the top 15 nodes based on CytoHubba.

Rank	Name
1	IL-10
1	IL-6
1	CXCL8
1	IL1B
5	VEGFA
6	ICAM1
6	CCL2
8	IFNG
9	CASP3
10	AKT1
11	IL2
12	IL4
13	CXCL10
14	MPO
15	SELE

### 3.6 Molecular docking

**FIGURE 10:**
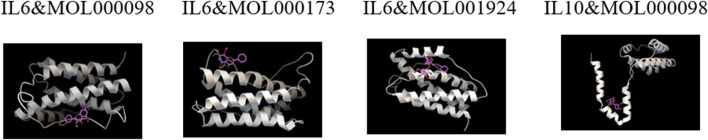
Representative molecular docking poses of WMF active compounds with IL-6 and IL-10. The 3D structures show the predicted binding modes of quercetin with IL-10, quercetin with IL-6, wogonin with IL-6, and paeoniflorin with IL-6.

Based on the network pharmacology results, three representative active compounds—quercetin (MOL000098), wogonin (MOL000173), and paeoniflorin (MOL001924) —were selected for docking against the hub targets IL-6 and IL-10. All three ligands dock efficiently into the binding pockets of both proteins (Figure 10). The lowest binding energies are summarized in Table 5. Quercetin binds IL-10 at −4.74 kcal/mol and IL-6 at −6.11 kcal/mol. Wogonin and paeoniflorin bind IL-6 with energies of −5.59 kcal/mol and −5.20 kcal/mol, respectively. These values indicate favorable interactions and support the predicted regulatory roles of these compounds in modulating IL-6/IL-10–mediated immune responses.

**TABLE 5:** Docking scores of the molecules.

Molecule name	Docking score (kcal/mol)
IL-10 and MOL000098 (quercetin)	−4.74
IL-6 and MOL000098 (quercetin)	−6.11
IL-6 and MOL000173 (wogonin)	−5.59
IL-6 and MOL001924 (paeoniflorin)	−5.20

### 3.7 Effects of WMF treatment on EAU *in vivo*

**FIGURE 11:**
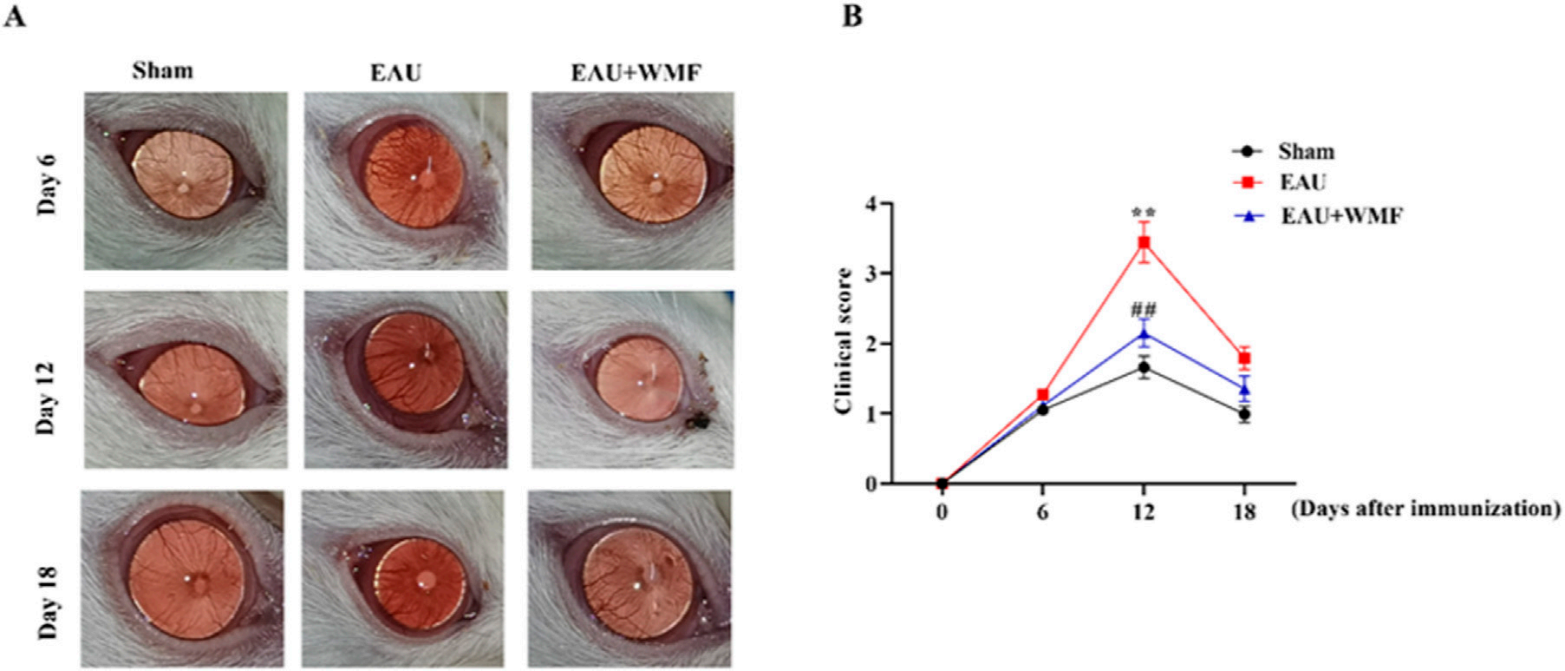
WMF ameliorates clinical signs of EAU in rats. **(A)** Representative slit-lamp photographs of the anterior segment. **(B)** Temporal changes in clinical scores. Data are presented as box plots (n = 8 rats per group). The overall effects of treatment, time, and their interaction on the ordinal clinical scores were analyzed using a Generalized Linear Mixed Model (GLMM). For comparisons at each individual time point, the Kruskal–Wallis H test was performed, followed by Dunn’s *post hoc* test for pairwise comparisons. **p < 0.01 vs Sham group; ##*p* < 0.01vs EAU group at the same time point.

**FIGURE 12:**
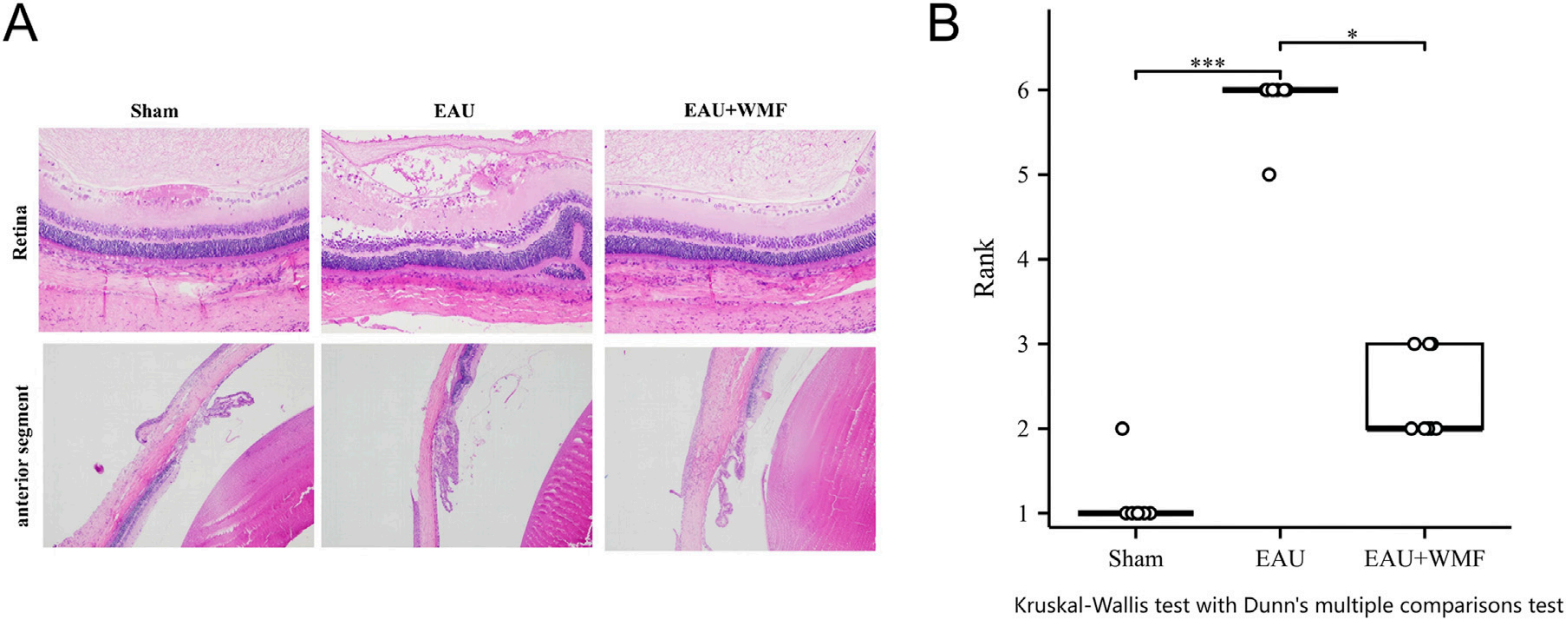
WMF treatment ameliorates retinal pathology in EAU rats. **(A)** Representative H&E-stained retinal sections from the Sham, EAU, and EAU + WMF groups. **(B)** Quantification of histopathological scores. The EAU group showed significantly increased scores compared to the Sham group (***p < 0.001), which were markedly reduced by WMF treatment (*p < 0.05). No significant difference was observed between the Sham and EAU + WMF groups. Data are shown as box plots (n = 8).

The EAU rat models were established to investigate the effects of WMF *in vivo*. Inflammation worsened in the EAU group from days 6–12, characterized by congestion and dilatation of the iris vessels and severe hypopyon in the anterior chamber after immunization (Figure 11). From days 12–18, these symptoms gradually improved. Compared to the control group, the inflammatory response in the EAU + WMF group was reduced, and clinical scores were lower. Hematoxylin and eosin (HE) staining revealed inflammatory cell exudation and infiltration, narrowing of the anterior chamber angle, and disorganization of the retinal layer structure in the retina and anterior segment of the EAU group. These pathological changes were significantly alleviated following WMF administration (Figure 12A) and confirmed by quantitative analysis of histopathological scores (Figure 12B).

**FIGURE 13:**
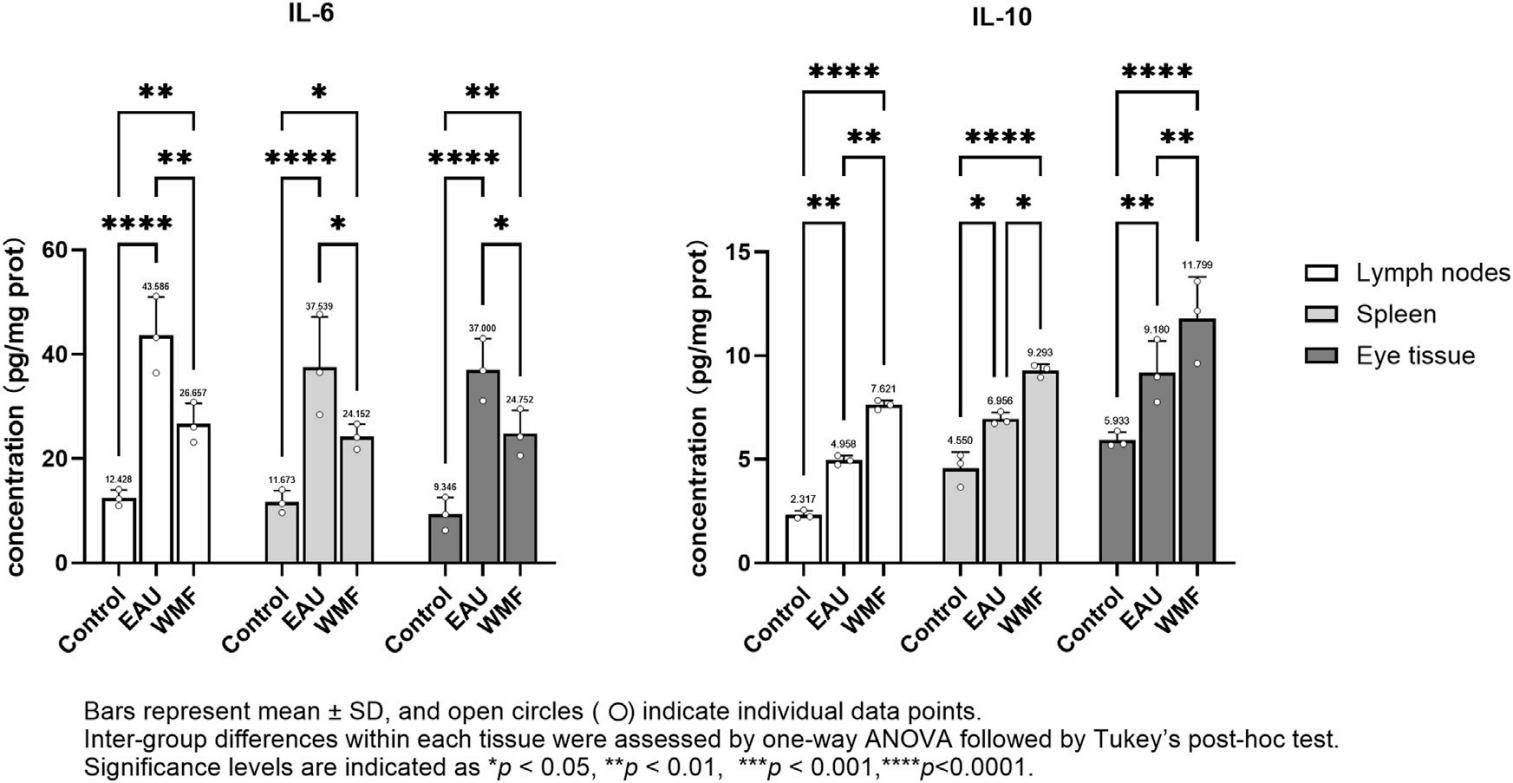
WMF modulates IL-6 and IL-10 protein concentrations in EAU rats, as determined by ELISA. Protein concentrations of IL-6 and IL-10 were measured in homogenates of lymph node, spleen, and ocular tissues at the experimental endpoint. Data are presented as column bars representing the mean, with T-error bars indicating the standard error (n = 3 composite samples per group, each measured in triplicate). Statistical significance between groups is indicated directly on the graphs (*p < 0.05, **p < 0.01, ***p < 0.001).

The levels of inflammatory cytokines, including IL-6 and IL-10, in the rat spleen, lymph nodes, and eye tissues were measured by ELISA. IL-6 concentration were significantly elevated in the EAU group across all three tissues; this elevation was reversed after WMF treatment (Figure 13). In contrast, IL-10 concentration were higher in the EAU group compared to the control group and increased further following WMF treatment (Figure 13). These findings suggest that WMF treatment modulates inflammatory responses by decreasing pro-inflammatory IL-6 levels while enhancing anti-inflammatory IL-10 levels.

**FIGURE 14:**
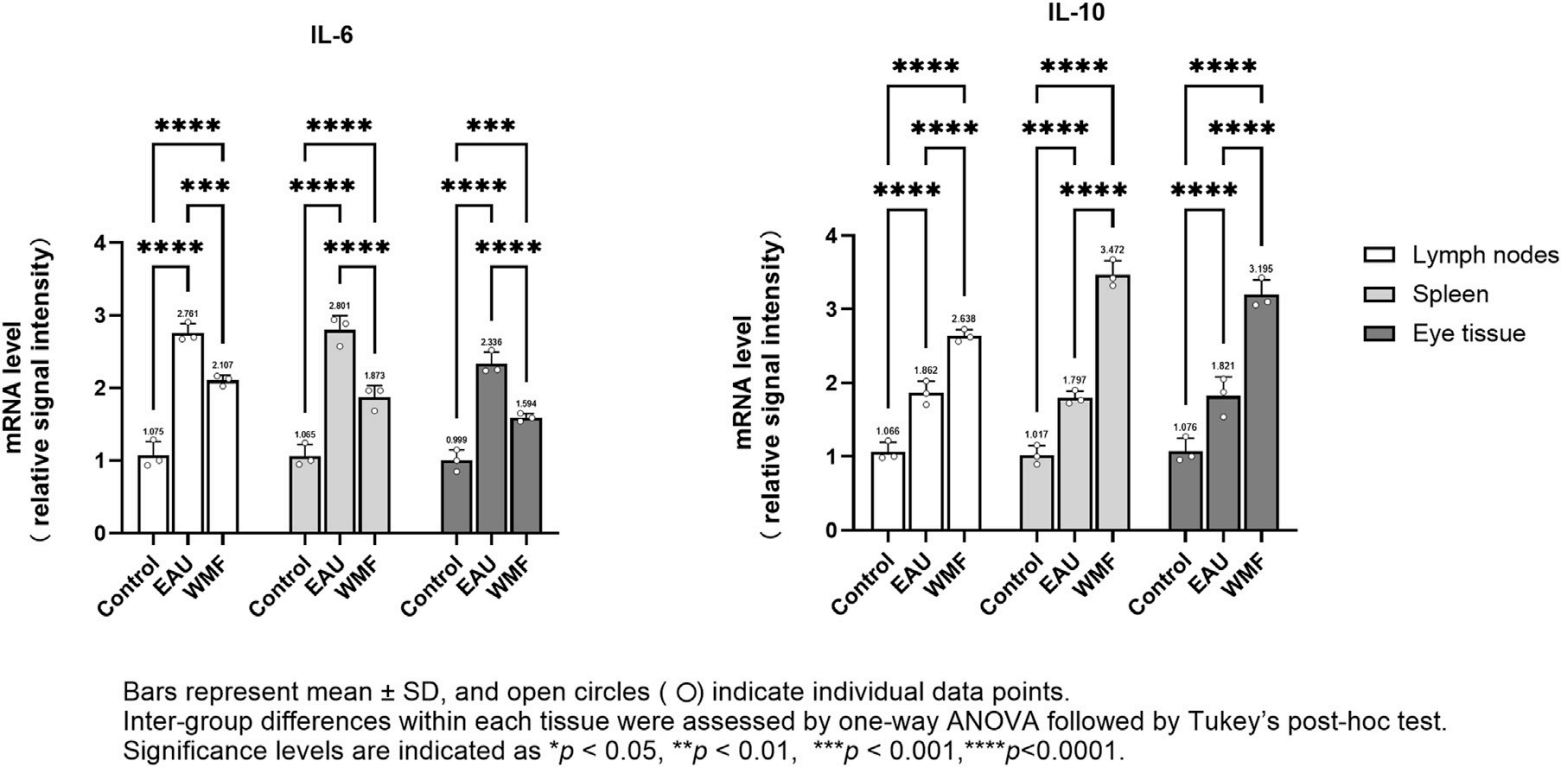
WMF regulates IL-6 and IL-10 mRNA expression levels in EAU rats, as determined by RT-qPCR. Relative mRNA expression levels of IL-6 and IL-10 were quantified using the 2^(−ΔΔCt)^ method with ACTIN as the internal reference. Data are presented as column bars representing the mean, with T-error bars indicating the standard error (n = 3 composite samples per group, each measured in triplicate). Statistical significance between groups is indicated directly on the graphs (*p < 0.05, **p < 0.01, ***p < 0.001).

**FIGURE 15:**
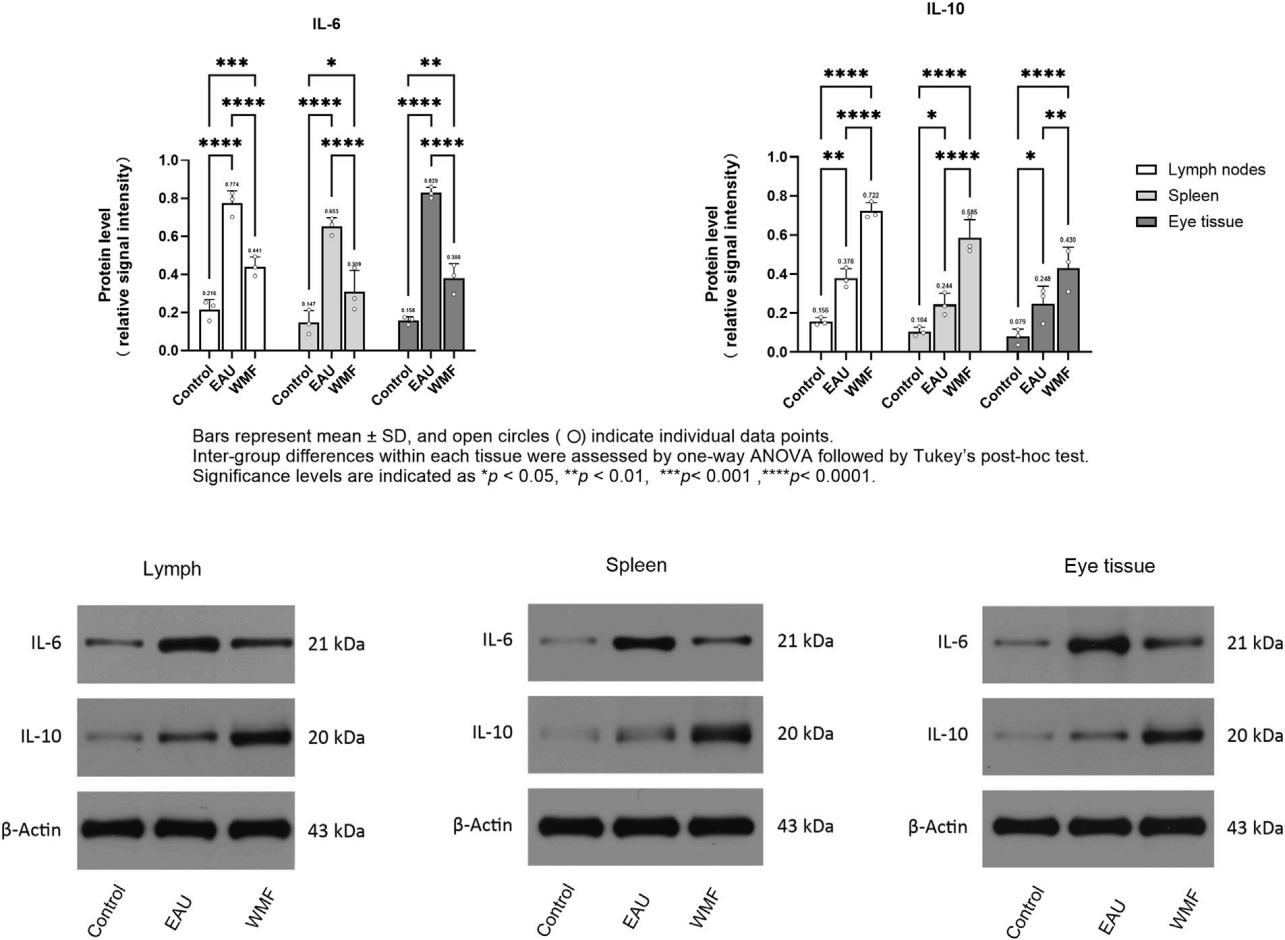
WMF alters IL-6 and IL-10 protein expression in EAU rats, as determined by Western blot analysis. Densitometric quantification of IL-6 and IL-10 protein levels normalized to β-Actin. Data are presented as column bars representing the mean, with T-error bars indicating the standard error (n = 3 composite samples per group). Statistical significance between groups is indicated directly on the graphs (*p < 0.05, **p < 0.01). Representative Western blot images for IL-6, IL-10, and β-Actin in lymph node, spleen, and ocular tissues.

Furthermore, RT-qPCR analysis showed that IL-6 expression was elevated in the lymph nodes, spleen, and eye samples of EAU model rats but decreased after WMF treatment (Figure 14). Similarly, IL-10 expression, which was increased in the EAU group, was further upregulated by WMF treatment (Figure 14). Western blot analysis corroborated these results, showing increased IL-6 expression in the lymph nodes of EAU rats that decreased in eye tissues following WMF treatment (Figure 15). IL-10 levels, elevated in the EAU group, were further increased by WMF treatment (Figure 15). Overall, these results indicated that WMF alleviated pathological changes and modulates the inflammatory response in EAU rats.

## 4 Discussion

Even in developed countries, Non-infectious uveitis accounts for 10%–15% of all cases of blindness, with a higher incidence in developing countries (Fan et al., 2021; Lin et al., 2014). Although corticosteroids have been used to treat NIU since the 1950s and multiple other drugs have been developed, corticosteroids remain the primary treatment for this disease (Woods, 1950; Gordon et al., 1951). Multisystem side effects occur with long-term corticosteroid administration despite the use of protective adjunct therapies (Ormaechea et al., 2019). TCM is effectively used in China to alleviate multisystemic side effects. However, the reliability of TCM for treating non-infectious uveitis validation through requires rigorous clinical and experimental studies. To address this need, bioinformatics methods can be used to improve the efficiency of such research.

In this study, a network diagram of active compounds, targets, and signaling pathways was constructed. We identified 16 active ingredients and 30 targets of WMF for treating EAU. GO enrichment analysis revealed that WMF could regulate the activation of multiple cytokines and receptor binding. KEGG enrichment analysis showed that WMF regulates multiple inflammation-related signaling pathways and vascular endothelial functions. PPI networks and critical subnetworks revealed 15 hub genes out of 30 genes. The core proteins IL-6 and IL-10 were selected for molecular docking with the related active components of WMF to verify the correlation. The bioinformatics results revealed that the WMF influenced the treatment of non-infectious uveitis and preliminarily elucidated its pharmacological mechanism of action. These findings can promote the development of new drugs for treating non-infectious uveitis.

This study primarily investigated the mechanism of action of WMF. To ensure clinical relevance, the animal experimental dose was derived from the clinical dose of WMF. Dose-response optimization was not included, as optimizing pharmacological efficacy was not the primary objective. This design rationale allowed us to focus on elucidating the mechanism of action of WMF while avoiding additional variables that could complicate the interpretation of the core mechanism.

Through bioinformatics analysis, we found that the herbal composition of WMF used for treating non-infectious uveitis could be simplified. The herbal composition of WMF was formulated under the guidance of TCM theory, and includes 5 Chinese herbs, *Juemingzi, Chishao, Cangzhu, Gouqizi, and Shayuanzi*. We analyzed the relationships among herbs, active compounds, target genes, and critical signaling pathways from a network pharmacology perspective. We found that 7 active components of SYZregulated 27 of the 30 therapeutic targets, including all 15 core proteins, and affect all 20 crucial signaling pathways. Based on network pharmacology analysis, SYZ may replace WMF to treat non-infectious uveitis. We also found that 3 active components of GQZ regulated 26 of the 30 therapeutic targets, including 14 of the 15 core proteins, and affect all 20 crucial signaling pathways. Thus, GQZ can also potentially substitute WMF for treating EAU. Additionally, 1 active component of CZ regulated 9 of the 30 therapeutic targets, including 5 of the 15 core proteins, and affect all 20 crucial signaling pathways. Therefore, CZ serves as an accessory herb in WMF for treating EAU. We found that 3 active components of JMZ regulated 5 of the 30 therapeutic targets, including 2 of the 15 core proteins, and affect all 20 crucial signaling pathways. Hence, JMZ is also an accessory herb in WMF for treating EAU. We found that 3 active components of CS regulated 10 of the 30 therapeutic targets, including 6 of the 15 core proteins, and affect 18 of the 20 crucial signaling pathways. Thus, CS is also an accessory herb in WMF for treating EAU. We speculate that SYZ or (and) GQZcould potentially substitute WMF to treat non-infectious uveitis; however further experimental studies are need to validate this speculation.

The results of the GO and KEGG analyses revealed that the selected targets were enriched primarily in inflammation-related immune regulation and vascular function. The biological processes of the target genes are consistent with the pathological mechanisms of non-infectious uveitis, including the regulation of T cells. EAU is an immune response mediated by T lymphocyte subsets and inflammatory cytokines. T-helper activation in immune-mediated uveitis involves three main pathways: (1) T-helper 1 response, characterized by the activation of IL-6, TNF-α, IFN-γ, and IL-2; (2)T-helper 2 response, characterized by the activation of IL-4, IL-5, IL-10, and IL-13, thus stimulating B-lymphocytes and antibodies production; and (3) T-helper 17 response, characterized by the activation of IL-17 and IL-23 (Caspi, 2003; Airody et al., 2016; Weinstein and Pepple, 2018; Karkhur et al., 2019; Guo and Zhang, 2021; Jung et al., 2019). The targets of WMF may regulate these three pathways. Subsequently, we applied the molecular docking techniques to validate the docking score of some active ingredients with the shared target proteinsIL-10 and IL-6. Our results showed that the active compounds of WMF effectively docked with IL-10 and IL-6. Furthermore, we established an EAU rat model to evaluate the therapeutic efficacy of WMF *in vivo* and found that WMF treatment significantly alleviated inflammation in EAU rats, reversed the EAU-induced increase in IL-6 levels, and increased IL-10 levels.

In this study, using a network pharmacology approach and molecular docking techniques, we investigated the molecular mechanisms underlying the therapeutic effects of WMF on EAU. The results of *in vivo* assays showed that WMF attenuated inflammation in EAU and regulated IL-10 and IL-6 expression in an EAU rat model. These findings indicate that WMF can treat EAU by modulating immune responses and vascular function, with IL-10 and IL-6 as the main therapeutic targets; moreover, the herbal composition can be further optimized based on these insights. We also found that some active substances, such as quercetin, may serve as effective drugs for treating EAU. However, in-depth mechanistic studies need to be performed for further experimental validation of our speculations.

To balance tissue requirements with statistical power, tissues from eight rats per group were pooled equally to generate three composite replicates. This approach maximized sample mass and minimized inter-assay variability, but obscured biological heterogeneity among individuals. Consequently, our data represent group-level averages and cannot infer inter-individual variability or extreme responders. Future work should incorporate independent biological replicates or single-animal analyses to quantify individual differences in treatment response and validate the generalizability of current findings.

## 5 Conclusion

We used network pharmacology and molecular docking methods to analyze the molecular mechanisms underlying the therapeutic effects of WMF on EAU. The results were verified through *in vivo* assays, which revealed that WMF can treat EAU by modulating immune processes and vascular functions. Additionally, we identified opportunities to optimize the herbal composition of WMF to enhance its therapeutic efficacy. These findings provide valuable insights into the treatment of EAU and suggest directions for future research.

## Data Availability

The original contributions presented in this study are included in the article/supplementary materials. Further enquiries may be directed to the corresponding authors.
